# MINFLUX nanoscopy enhanced with high-order vortex beams

**DOI:** 10.1038/s41377-025-01822-0

**Published:** 2025-05-06

**Authors:** Xiao-Jie Tan, Zhiwei Huang

**Affiliations:** 1 Optical Bioimaging Laboratory, Department of Biomedical Engineering, College of Design and Engineering, National University of Singapore, Singapore, 117576 Singapore; 2 National University of Singapore (Suzhou) Research Institute, Suzhou, Jiangsu 215123 China; 3 NUS Graduate School for Integrative Sciences and Engineering Programme (ISEP), National University of Singapore, Singapore, 119077 Singapore

**Keywords:** Biophotonics, Super-resolution microscopy

## Abstract

Minimal photon fluxes (MINFLUX) nanoscopy has emerged as a transformative advancement in superresolution imaging, enabling unprecedented nanoscale observations across diverse biological scenarios. In this work, we propose, for the first time, that employing high-order vortex beams can significantly enhance the performance of MINFLUX, surpassing the limitations of the conventional MINFLUX using the first-order vortex beam. Our theoretical analysis indicates that, for standard MINFLUX, high-order vortex beams can improve the maximum localization precision by a factor corresponding to their order, which can approach a sub-nanometer scale under optimal conditions, and for raster scan MINFLUX, high-order vortex beams allow for a wider field of view while maintaining enhanced precision. These findings underscore the potential of high-order vortex beams to elevate the performance of MINFLUX, paving the way towards ultra-high resolution imaging for a broad range of applications.

## Abstract (web summary)

High-order vortex beams are proven to enable the enhanced performance of MINFLUX nanoscopy, opening a new window for ultra-high-resolution imaging across diverse applications.

## Graphical abstract

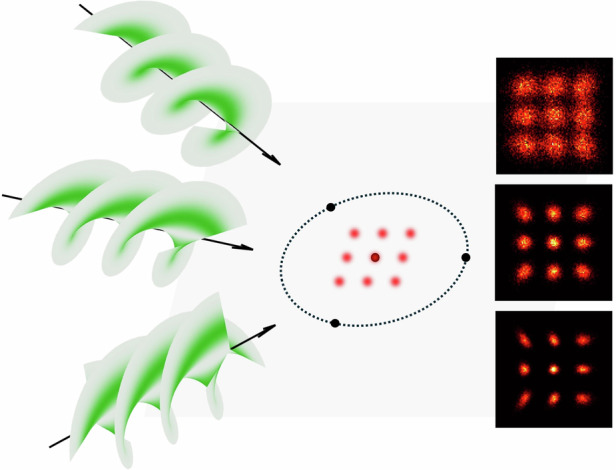

## Introduction

Pursuing resolution beyond the diffraction limit of conventional optical systems has become pivotal in contemporary biomedical research. Over the past few decades, several superresolution methods have been developed to surpass the diffraction limit, such as stimulated emission depletion (STED) microscopy^1^, photoactivated localization microscopy (PALM)^2^ and stochastic optical reconstruction microscopy (STORM)^3^. Notably, minimal photon fluxes (MINFLUX) nanoscopy^4^, proposed in 2017 by Balzarotti et al., marks a transformative advancement in fluorescence imaging, achieving unprecedented spatial resolution down to the nanometer scale. By employing a doughnut-shaped excitation beam featuring a central dark point to scan the sample, MINFLUX allows for the precise localization of single emitters with fewer photons, thereby significantly reducing photon flux and preventing potential photobleaching or phototoxicity. When combined with controllable fluorescence excitation, MINFLUX has delivered exceptionally high resolution for the observation of various biological samples and processes, facilitating a deeper understanding of cellular dynamics, protein interactions, and complex biological mechanisms at a molecular level^5–17^.

While the original MINFLUX typically employs a four-point targeted coordinate pattern (TCP) and a first-order vortex beam as the doughnut, alternatives have been developed to enhance its performance. Masullo et al.^18^ introduced raster scanning MINFLUX (RASTMIN) to reduce the experimental complexity. It is demonstrated that RASTMIN delivers performance comparable to MINFLUX, with a simplified implementation which is more compatible with confocal scanning microscopes. Zhao et al.^19^ proposed that two-photon fluorescence^20^ improves the precision of MINFLUX. By exciting two-photon fluorescence, the optimal localization precision is doubled compared with the single-photon case. Recently in Ref. ^21^, the performance of multiphoton MINFLUX and RASTMIN has been theoretically analyzed.

Multiphoton luminescence^22^ enhances the performance of MINFLUX essentially by engineering the point spread function (PSF). Near the central area of the field of view (FOV), this optimization is analogous to utilizing a high-order (HO) vortex beam. Moreover, HO vortex beams offer greater degrees of freedom and better compatibility for deployment, eliminating the need for modifications to either the laser source or the samples. Nevertheless, a systematic analysis of HO vortex beam based MINFLUX remains unexplored, leaving a critical gap that impedes the full exploitation of the potential of MINFLUX.

In this work, we theoretically demonstrate that the performance of MINFLUX can be significantly enhanced by employing HO vortex beams as excitation. Compared to the conventional MINFLUX with the first-order vortex beam, HO vortex beams offer superior precision for localizing single emitters. For MINFLUX with conventional four-point TCP, the central precision increases proportionally to the vortex order, indicating a substantially improved precision for iterative measurements, while for raster scanning MINFLUX, enhanced precision is achieved across a broader area, suggesting an extended FOV for fixed measurements.

## Results

### Principle of HO MINFLUX

Conventional MINFLUX generally utilizes a first-order vortex beam as the doughnut excitation, which is a special type of Laguerre-Gaussian (LG) beam^23^. The amplitude profile of a Laguerre Gaussian beam is given by

1 $${\varphi }_{lp}({\boldsymbol{r}})=\sqrt{\frac{2p!}{\pi (p+|l|)!}}\frac{1}{w}{\left(\frac{\sqrt{2}r}{w}\right)}^{|l|}{L}_{p}^{|l|}\left(\frac{2{r}^{2}}{{w}^{2}}\right){e}^{-\frac{{r}^{2}}{{w}^{2}}}{e}^{-il\theta }$$

where ${\boldsymbol{r}}\,=\,(r,\theta )$ is the position vector in the polar coordinate system, *l* and *p* are the azimuthal and radial indices, respectively. *w* is the radius of beam waist, which is related to the full width at half maximum (FWHM) as $\text{FWHM}=\sqrt{2\,\mathrm{ln}\,2}w$. ${L}_{p}^{|l|}(\bullet )$ is the associated Laguerre polynomial.

When $l\,\ne\, 0$, the LG beam exhibits vortex phase front and carries orbital angular momentum (OAM)^24,25^, where the center of the beam appears as the singularity, presenting a doughnut-like profile. The order of vortex is determined by the azimuthal index *l*, also known as the topological charge. *l* = 1 refers to the most widely applied doughnut beam. The radial index *p* brings more oscillations to the beam profile. As the region of interest (ROI) of MINFLUX is typically much smaller than the beam width, the *p* index has negligible effect on the beam profile as well as the precision of MINFLUX, as will be elaborated on later. Therefore, in this study, we mainly focus on cases that *p* = 0.

The intensity profile of an *l*-order vortex beam is described by

2 $$I({\boldsymbol{r}})\propto {\left(\frac{2{r}^{2}}{{w}^{2}}\right)}^{|l|}{e}^{-\frac{2{r}^{2}}{{w}^{2}}}$$

As can be noted, in the vicinity of central region, $I({\boldsymbol{r}})\propto {r}^{2|l|}$, which behaves similarly to the PSF of $|l|$-photon fluorescence when using a first-order vortex beam. Thus, while multiphoton excitation has been shown to improve the localization precision of MINFLUX^19,21^, HO vortex-based MINFLUX achieves comparable performance and offers a more convenient implementation, requiring only the modulation of the excitation wavefront.

**Fig. 1:**
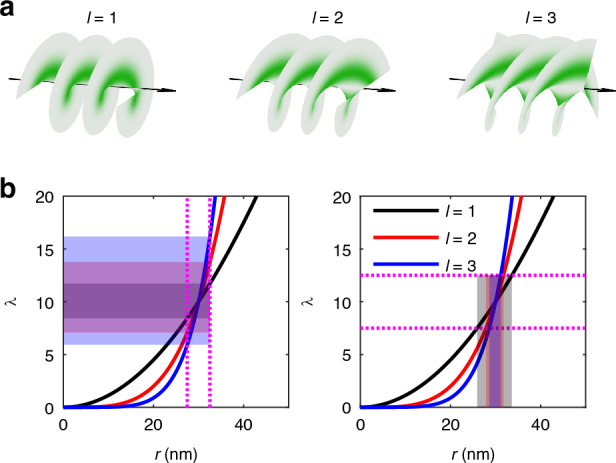
Enhanced localization precision with HO vortex beams. **a** Helical wavefronts of vortex beams with different orders. **b** Illustration of enhanced sensitivity (left) and reduced uncertainty (right) achieved using HO vortex excitation. Shaded regions represent the variations in $\lambda (r)$ with respect to $r(\lambda )$. Beam radius *w* is set to 300 nm

The enhancement of localization precision with HO vortex beams can be intuitively illustrated by the sensitivity of photon detection on the position, or the propagation of uncertainty from photon detection to position estimation, as is shown in Fig. 1. Considering the arrival of photons follows a Poisson process, the Poisson rate $\lambda$, which represents the mean photon count, is proportional to the excitation intensity, $\lambda \propto I(r)$. We can then derive that

3 $$\frac{\partial \lambda }{\partial r}=\frac{2\lambda }{r}\left(|l|-\frac{2{r}^{2}}{{w}^{2}}\right)$$

Near the center where $r\ll w$, we have $\frac{\partial \lambda }{\partial r}\approx \frac{2\lambda }{r}|l|$. Consequently, given the same position $r$ and Poisson rate $\lambda$, the photon counts detected in HO vortex are more sensitive to the variation of position. Regarding the propagation of uncertainty, with the same uncertainty in photon detection, the uncertainty in position estimation is decreased for HO vortex beam. For an *l*-order vortex beam, there is an $|l|$-fold increase in the sensitivity and decrease in the uncertainty, which enhances the performance in localization microscopy.

### Localization precision analysis

To see how the order of vortex beams exactly affects the MINFLUX precision of localization, we analyze the Cramér-Rao Bound (CRB) for different order vortex beams and the effect of some other practical parameters, such as the length of TCP *L* and the signal to background ratio (SBR).

**Fig. 2:**
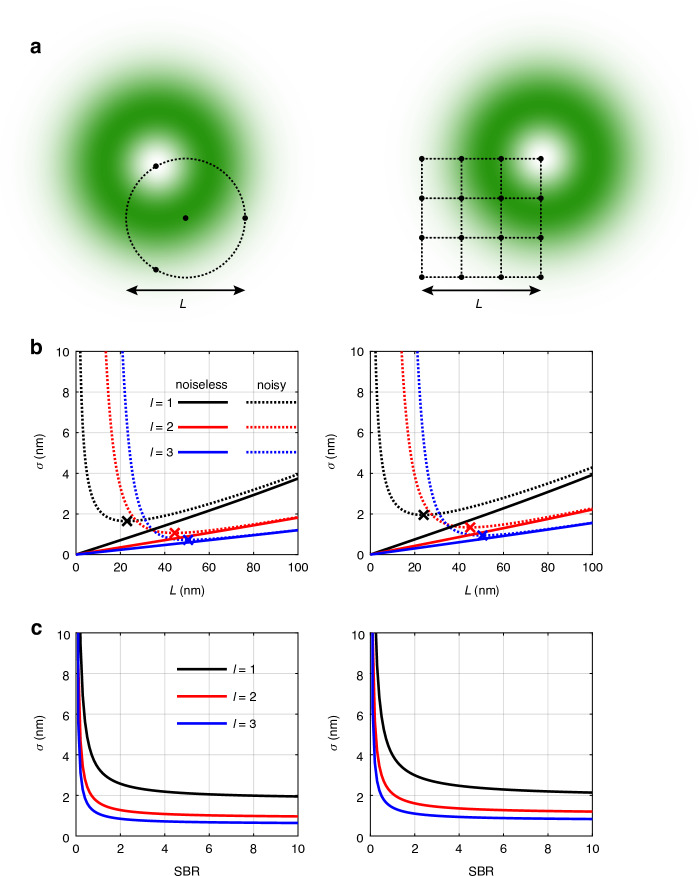
Performance of MINFLUX with HO vortex beam. **a** The four-point TCP and raster scan TCP. **b** Central CRB plotted as a function of *L*. For noisy cases, SBR is set as 4 at *L* = 50 nm. **c** Central CRB plotted as a function of the SBR, where $L={\rm{50nm}}$. The total photon count is set as 100

Generally, two common TCPs are employed in MINFLUX: the widely used four-point TCP and the raster scan TCP, as illustrated in Fig. 2a. We therefore examine both configurations for comparison.

The analytical expression for the CRB of MINFLUX is typically complex, influenced by the illumination pattern, the TCP and the position of the emitter, see Method for the detailed derivations. For conventional MINFLUX with four-point TCP, an explicit expression can be derived for the central CRB as

4 $${\sigma }_{{\bf{0}}}=\frac{L}{2n\sqrt{2N}}\frac{s}{|l|-\frac{{L}^{2}}{2{w}^{2}}}$$

Here *n* stands for the *n*-photon excitation, *l* refers to the order of vortex beam, *N* is the number of detected photons and $s=\sqrt{(1+\frac{3}{4\text{SBR}})(1+\frac{1}{\text{SBR}})}$. Equation (4) is a generalization of Eq. (2) in Ref. ^19^. Apparently, the order *l* improves the central CRB slightly more than $|l|$-fold, as *L* is much smaller than *w*. Vortex order *l* thus plays a similar role as *n*, requiring only $1/{l}^{2}$ of the photons to achieve the same precision as a first order doughnut beam. The excitation wavelength primarily influences the precision of MINFLUX by affecting the focused beam size *w*, which has a negligible impact in the subdiffraction region, as studied in Ref.^4,19^.

To facilitate a comparison between MINFLUX and RASTMIN, we numerically calculate the CRB for both TCPs. Figure 2b illustrates the central CRB as a function of *L*. In the excess noise-free scenario represented by the solid curves, the CRB always decreases as *L* reduces, with higher-order vortex beams consistently yielding smaller CRBs. In this ideal case, higher-order vortices always achieve superior precision given the same *L*. For noisy scenarios, as represented by the dashed curves, the presence of background noise significantly affects the localization precision of MINFLUX, as the signal can be overwhelmed by the noise near the center. Consequently, continuously decreasing *L* gives rise to a very low SBR, which may lead to a dramatic increase in the CRB. An optimal *L* which minimizes the CRB can be identified for each vortex beam, as indicated by the crosses on the dashed lines in Fig. 2b. The minimum CRBs achieved under MINFLUX for *l* = 1, 2, and 3 are 1.64 nm, 1.06 nm, and 0.72 nm, respectively, while under RASTMIN, the corresponding values are 1.95 nm, 1.34 nm, and 0.94 nm, respectively. For both TCPs, higher-order vortices deliver superior optimal precisions.

It should be noted that the SBR for HO vortex beams is more sensitive. For instance, in MINFLUX, the relation between SBR and *L* is given by

5 $${\rm{SBR}}_{L}={\text{SBR}}_{{L}_{0}}{\left(\frac{L}{{L}_{0}}\right)}^{2|l|}{e}^{-\frac{{L}^{2}-{L}_{0}^{2}}{2{w}^{2}}}$$

where the SBR decreases more rapidly for HO vortex beams as *L* reduces. Therefore, at small *L*, HO vortex beams may result in even larger errors due to the extremely low SBR.

Figure 2c analyzes the effect of SBR, where the length of TCP is set to be 50 nm. It is evident that when the SBR is too low (e.g. SBR < 1), the CRB becomes substantially large, impeding the precise localization in MINFLUX. Improving the SBR significantly reduces the CRB, which eventually converges to the precision in noiseless conditions.

While in both MINFLUX and RASTMIN, HO vortex beams improve the maximum precision at the center, MINFLUX with four-point TCP offers superior improvement. For example, with *L* = 50 nm, SBR = 4, central CRB values under MINFLUX for *l* = 1, 2, 3 are 2.31 nm, 1.15 nm and 0.76 nm, respectively. This improvement slightly exceeds *l*-fold, as can also be inferred from Eq. (4). In RASTMIN, the corresponding values are 2.66 nm, 1.44 nm, and 1.00 nm, showing a smaller improvement on the central CRB compared to MINFLUX. Compared with several variants of MINFLUX which have demonstrated nanometer-scale resolution (as summarized in the table in Ref.^26^), HO vortex beams hold significant promise for further improving MINFLUX precision to the sub-nanometer level under optimized conditions.

**Fig. 3:**
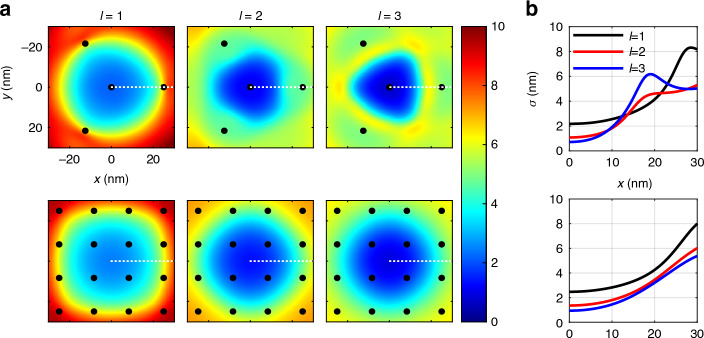
The CRB for vortex beams with order l = 1, 2, 3. **a** CRB distribution over 2D space. **b** Variation of CRB along *x* axis as indicated by the dashed lines in **a**. Top: four-point TCP. Bottom: raster scan TCP. Parameter setting: *N* = 100, *L* = 50 nm, SBR = 4

While MINFLUX excels in achieving higher central precision, RASTMIN offers advantages with its expanded FOV. In Fig. 3a, we study the CRB distribution across two-dimensional (2D) space. As can be observed, for MINFLUX, increasing the vortex order significantly reduces the central CRB, but this improvement is limited to the central region. The CRB deteriorates considerably outside the central area, indicating that optimal localization requires confining the emitter near the center of the TCP. In contrast, RASTMIN, despite its slightly inferior central CRB compared to MINFLUX, enhances the CRB more uniformly across the entire FOV, accommodating a larger region for the emitter. Therefore, MINFLUX is particularly well-suited for an iterative scan scheme, which progressively confines the emitter towards the center of the TCP, while RASTMIN is more effective with a fixed scan scheme, providing a larger FOV for tracking emitters over extended areas. Figure 3b plots the CRB along 0 degree for different orders of vortex beams. In MINFLUX, the CRB improves significantly with HO vortex beams when the emitter is near the central region. However, the precision may even drop below that of lower-order beams when the emitter deviates further from the center. For RASTMIN, the CRB is improved for HO vortex within the entire FOV. Additionally, the increase in CRB from the center to the edge is slower in RASTMIN compared to MINFLUX, indicating more uniform performance across the FOV.

Define the effective FOV (EFOV) as the circular area where the CRB is below 4 nm, a threshold chosen to represent high-precision localization capabilities. Under this definition, we quantify the EFOV for different orders of vortex beams in both MINFLUX and RASTMIN. For MINFLUX, the diameter of EFOV is 35.85 nm for *l* = 1, 32.80 nm for *l* = 2, and decreases to 29.19 nm for *l* = 3. For RASTMIN, the EFOV shows an evident increase, from 37.88 nm for *l* = 1, to 42.80 nm for *l* = 2, and 45.93 nm for *l* = 3. These data intuitively show that increasing the vortex order *l* significantly expands the EFOV in RASTMIN, suggesting the potential for imaging larger areas with high precision, which could be particularly beneficial for studying extended cellular structures or dynamic processes over wider regions.

In practice, prior information about emitter’s position is often available through approaches such as wide-field imaging, preliminary confocal scans, or initial rounds of conventional MINFLUX. Under such conditions, the Bayesian CRB^27,28^ can be used to evaluate the precision of localization. Typically, prior information yields a normal distribution for the emitter’s position^29^. Assuming a standard deviation of 50 nm for the prior distribution, the Bayesian CRB for MINFLUX is calculated to be 6.95 nm, 4.59 nm, 3.63 nm for *l* = 1, *l* = 2, and *l* = 3, respectively. For RASTMIN, the corresponding values are 7.22 nm, 4.80 nm, and 3.81 nm. Both cases show that the precision conditioned on prior information can be significantly improved with HO vortex beams.

Besides the improved precision, another advantage of HO vortex beams lies in their enhanced robustness within turbid scattering media, such as biological tissues. As demonstrated in previous works^30,31^, higher-order vortex beams exhibit higher transmittance in strongly scattering environments, enabling them to penetrate deeper into biological tissues. This property further enhances the capability of HO vortex beams for deep in vivo biological observations.

### The effect of radial index

In the previous section, we examined the performance of MINFLUX for the case where *p* = 0. Here, we show that the radial index *p* has a negligible effect on the localization precision of MINFLUX.

According to Eq. (1), the radial index *p* influences the beam profile via the associated Laguerre polynomial. Since the ROI in MINFLUX is typically much smaller than the beam width, the leading term of the associated Laguerre polynomial in this case is a constant. As a result, the profile of the beam within the ROI is not significantly affected by the radial index, which in turn subtly impacts the localization precision of MINFLUX.

**Fig. 4:**
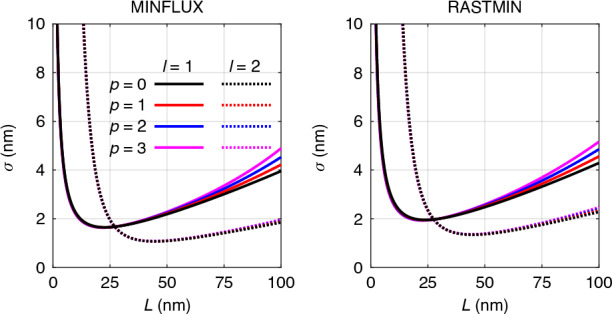
The effect of radial index on the central CRB. Left: MINFLUX. Right: RASTMIN. Different line styles represent different *l* index, while different colors denote different *p* index. Parameter setting: *N* = 100, SBR = 4

In Fig. 4, we numerically calculate the central CRB for vortex beams with different radial indices. It is apparent that for both MINFLUX and RASTMIN, beams with the same azimuthal index (solid and dashed curves) yield nearly identical CRB values, irrespective of the radial index. Noticeable divergence in CRB values emerges when the length of the TCP is excessively large, and this divergence reduces as the azimuthal index *l* increases. The 2D CRB map for different radial indices is presented in Supplementary Section I, where it is also observed that the CRB within the entire ROI changes little with varying *p* indices.

### Monte Carlo simulation

To verify our theoretical analysis and demonstrate the performance of HO vortex-based MINFLUX, we employ Monte Carlo simulation method to simulate the localization process in MINFLUX. We generate multinomial random numbers to represent photon counts at each scanning point, corresponding to the probability distribution over the TCP. Subsequently, we apply maximum likelihood estimation (MLE) to retrieve the emitter’s position. The estimator used in MLE is given by

6 $${\hat{{\boldsymbol{r}}}}_{e}=\mathop{\text{arg}\,\max }\limits_{{{\boldsymbol{r}}}_{e}}P({\boldsymbol{n}}|{{\boldsymbol{r}}}_{e})$$

**Fig. 5:**
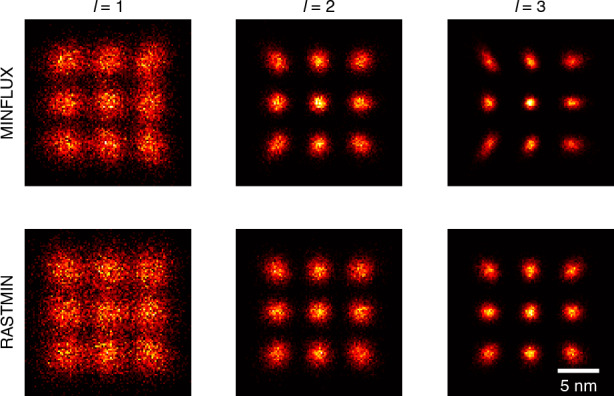
Simulated results for HO vortex based MINFLUX localization. Top: MINFLUX, bottom: RASTMIN. Scale bar: 5 nm. Simulation conditions: *N* = 300, L = 50 nm, SBR = 4, trials = 2000

Details refer to Methods section. We conduct Monte Carlo simulations for both MINFLUX and RASTMIN using different orders of vortex beams, with results summarized in Fig. 5. The sample considered here is a 3 × 3 array of emitters, which can be realized using fluorophore-labeled DNA origami. The distance between adjacent emitters is 5 nm. In each trial, we set the total photon number *N* to 300, the length of TCP *L* to 50 nm, and the SBR to 4. For each case, 2000 trials are performed to evaluate the root mean square error (RMSE), which is defined as

7 $${\text{RMSE}}=\sqrt{\frac{1}{2}{\rm{E}}({|{{\hat{\boldsymbol{r}}}}_{e}-{{\boldsymbol{r}}}_{e}|}^{2})}$$

where the factor 2 is to keep consistency with the arithmetic mean of CRB in Eq. (16).

The results demonstrate that HO vortex beams significantly enhance the resolvability of the emitters, achieving substantially smaller estimation errors compared to conventional MINFLUX. The average localization error decreases from 1.33 nm (*l* = 1) to 0.75 nm (*l* = 2) and 0.59 nm (*l* = 3). Comparable improvements are observed in RASTMIN, with the average error reduced from 1.52 nm to 0.88 nm and 0.64 nm, respectively. The simulations also validate the effectiveness of the CRB derived in this study. The overall RMSEs for all cases remain within 5% of the theoretical bounds, illustrating that the localization precision is well-bounded by the CRB.

Additionally, as originally noted by Balzarotti et al.^4^, the localization precision is not isotropic in 2D space. Our simulations also reveal that the estimated positions are not isotropically distributed along each direction when the emitter is not at the center of TCP. While the estimated positions generally follow bivariate Gaussian distributions, their standard deviations along orthogonal axes are typically unequal, except for the central emitter. This anisotropy becomes more noticeable with the use of HO vortex beams, particularly in MINFLUX. For instance, in 3rd-order MINFLUX, the estimated positions of edge emitters exhibit strong anisotropy. The results in RASTMIN also indicate anisotropy, although the effect is less pronounced compared to MINFLUX. A detailed analysis of the anisotropy of CRB is provided in Supplementary Section II.

## Discussion

Through the analysis presented, we demonstrate that HO vortex beams can enhance the performance of MINFLUX in both conventional and raster scan TCP modes, each with distinct advantages. For conventional MINFLUX, an *l*-order vortex beam improves the maximum precision by a factor of *l*, enabling ultra-high achievable resolution under optimal conditions. However, this improvement in precision is primarily confined to the central region, making it better suited for iterative measurements. For RASTMIN, although the increase in maximum precision is not as pronounced as in MINFLUX, the improvement extends across a larger FOV with a more isotropic and uniform distribution, which allows for highly precise localization of samples over a broad range in fixed observations.

In practice, the precision of MINFLUX is predominantly limited by noise, particularly for HO vortices. As demonstrated in Supplementary Section III, HO vortices exhibit higher sensitivity to background noise compared to their lower-order counterparts. As background intensity increases, the precision of HO vortices is more severely impacted due to the rapid decline in the SBR, thereby hindering the broader utilization of HO vortices in MINFLUX applications. This issue poses a significant challenge for advancing HO vortex beam-based MINFLUX, where emissions from the large central dark region could be overwhelmed by noise, impeding the extraction of sufficient signals for accurate localization. Maintaining a high SBR for HO vortex beams requires stringent experimental conditions, such as highly sensitive single photon detectors with negligible dark counts and efficient optical filters to eliminate excitation and ambient light. Other experimental imperfections, including system vibrations and aberrations^32^, should also be compensated accordingly.

The generation of HO vortex beams has been extensively studied using various methods, including wave plates^33^, spatial light modulators^34^, metamaterials^35^, fibers^36^ or directly from laser cavities^37^. For seamless integration with conventional MINFLUX setup, HO vortex phase plate (VPP)^38^ can be considered as a straightforward replacement of the standard 2π VPP. When the same circularly polarized light is transmitted through a HO VPP and subsequently focused by a high numerical aperture (NA) objective lens, a tightly focused HO vortex beam can be efficiently produced. A numerical demonstration, provided in Supplementary Section IV, offers more quantitative analysis of the beam characteristics.

Future studies for the optimization of MINFLUX can be expanded in various directions. For example, three-dimensional (3D) enhanced MINFLUX can be achieved by further modulating the axial profile of the 3D optical doughnut. Meanwhile, more degrees of freedom, such as polarization^39^, can be considered for improving the performance of MINFLUX. In addition, integrating MINFLUX with other label-free methods^40^ offers complementary advantages. In complex scenarios, multimodal imaging incorporating fluorescence and non-fluorescence techniques facilitates resolving samples at different scales, enabling superresolution across a wide range of applications.

More broadly, investigating the optimal light beam or modulation method for superresolution in different imaging modalities remains an intriguing topic. Delving into effective modulation strategies for various scenarios merits both theoretical and experimental efforts, holding great potential for advancing diverse biomedical studies.

## Materials and methods

The CRB of MINFLUX can be derived as follows. Consider *n*-photon fluorescence, the intensity of detected emission is proportional to the *n-*th power of the intensity of the excitation light,

8 $${\lambda }_{k}=\beta {I}^{n}({{\boldsymbol{r}}}_{e}-{{\boldsymbol{r}}}_{k})$$

where ${{\boldsymbol{r}}}_{e}$ is the position of the emitter and $\{{{\boldsymbol{r}}}_{k}\}$ represents the TCP of the excitation. $\beta$ is a coefficient representing the effect of collection efficiency of the system, quantum yield and absorption cross-section of the fluorophore.

The probability distribution of detected photons normalized over the TCP is thus given by

9 $${p}_{k}=\frac{{\lambda }_{k}}{\mathop{\sum }\nolimits_{k=1}^{K}{\lambda }_{k}}$$

In the presence of background noise, the probability distribution is accordingly modified as

10 $${p}_{k}=\frac{{\lambda }_{k}+{\xi }_{k}}{\mathop{\sum }\nolimits_{k=1}^{K}({\lambda }_{k}+{\xi }_{k})}$$

where ${\xi }_{k}$ is background intensity at ${{\boldsymbol{r}}}_{k}$.

The signal to background ratio is then defined as

11 $${\text{SBR}}=\frac{\mathop{\sum }\nolimits_{k=1}^{K}{\lambda }_{k}}{\mathop{\sum }\nolimits_{k=1}^{K}{\xi }_{k}}$$

For simplicity, we assume a uniform background across all scanning positions, i.e. ${\xi }_{k}=\xi$ for every *k*.

It should be noted that SBR is influenced by the TCP as well as the emitter. In MINFLUX and RASTMIN, SBR is therefore a function of the TCP length *L* and the emitter’s position ${{\boldsymbol{r}}}_{e}$. In this study, the SBR is generally set with reference to a central emitter and a specific length.

Given a total of *N* collected photons, the probability distribution of photon detection across different positions follows a multinomial distribution,

12 $$P({\boldsymbol{n}}|{{\boldsymbol{r}}}_{e})=\frac{N!}{\mathop{\prod }\nolimits_{k=1}^{K}{n}_{k}!}\mathop{\prod }\limits_{k=1}^{K}{p}_{k}^{{n}_{k}}$$

where ${\boldsymbol{n}}=({n}_{1},{n}_{2},\cdots ,{n}_{K})$, and ${n}_{k}$ is the number of photons detected at ${{\boldsymbol{r}}}_{k}$ satisfying $N={\sum }_{k}{n}_{k}$.

The score function is expressed as

13 $${\boldsymbol{S}}=\nabla \,{\mathrm{ln}}\,P({\boldsymbol{n}}|{{\boldsymbol{r}}}_{e})=\mathop{\sum }\limits_{k=1}^{K}{\left[\frac{{n}_{k}}{{p}_{k}}\frac{\partial \,{p}_{k}}{\partial x},\frac{{n}_{k}}{{p}_{k}}\frac{\partial {p}_{k}}{\partial y}\right]}^{T}$$

We can then derive the Fisher information matrix as

14 $${{\boldsymbol{J}}}_{{\boldsymbol{r}}}=E({\boldsymbol{S}}{{\boldsymbol{S}}}^{T})=N\mathop{\sum }\limits_{k=1}^{K}\frac{1}{{p}_{k}}\left[\begin{array}{cc}{\left(\frac{\partial {p}_{k}}{\partial x}\right)}^{2} & \frac{\partial {p}_{k}}{\partial x}\frac{\partial {p}_{k}}{\partial y}\\ \frac{\partial {p}_{k}}{\partial y}\frac{\partial {p}_{k}}{\partial x} & {\left(\frac{\partial {p}_{k}}{\partial y}\right)}^{2}\end{array}\right]$$

The lower bound of the covariance matrix, represented as the Cramér-Rao bound, is given by

15 $${{\boldsymbol{\Sigma }}}_{{\boldsymbol{r}}}={{\boldsymbol{J}}}_{{\boldsymbol{r}}}^{-1}$$

Following convention, we primarily investigate the arithmetic mean of the eigenvalues of ${{\boldsymbol{\Sigma }}}_{{\boldsymbol{r}}}$,

16 $${\sigma }_{{\boldsymbol{r}}}=\sqrt{\frac{1}{2}\text{Tr}{{\boldsymbol{\Sigma }}}_{{\boldsymbol{r}}}}$$

In this context, the term CRB explicitly refers to ${\sigma }_{{\boldsymbol{r}}}$ in Eq. (16).

## Supplementary information

Supplementary Information

## Data Availability

Data underlying the results presented in this paper are not publicly available at this time but may be obtained from the corresponding author upon reasonable request.
