# Supplementary material for: MINFLUX nanoscopy enhanced with high-order vortex beams
Source: Light Sci Appl. 2025 May 6;14:184. doi: 10.1038/s41377-025-01822-0 (PMC12052830; doi:10.1038/s41377-025-01822-0)
Supplement: Supplementary file 1 — Supplementary Information [file 41377_2025_1822_MOESM1_ESM.pdf]

Supplementary Information for

MINFLUX Nanoscopy Enhanced with High-Order Vortex Beams

Xiao-Jie Tan[1] and Zhiwei Huang[1, 2, 3, *]

[1] Optical Bioimaging Laboratory, Department of Biomedical Engineering, College of Design and Engineering, National University of Singapore, Singapore 117576

[2] National University of Singapore (Suzhou) Research Institute, Suzhou, Jiangsu 215123, China

[3] NUS Graduate School for Integrative Sciences and Engineering Programme (ISEP), National University of Singapore, Singapore 119077

[*] Correspondence: biehzw@nus.edu.sg

I. CRB with different radial indices

In the main text, we have analyzed the effect of the radial index p and inferred that it would not significantly affect the performance of MINFLUX. Here we provide a more detailed analysis of the radial indices.

Let $t = 2r^2/w^2$; the intensity of the LG beam with a nonzero p index is given by

$$I(t) \propto (t)^{|l|} e^{-t} \left[L_p^{|l|}(t) \right]^2 . \tag{S1}$$

Within the ROI of MINFLUX where $r \ll w$, the associated Laguerre polynomial is primarily dominated by its constant term,

$$L_p^{|l|}(t) = \sum_{k=0}^{p} (-1)^k \binom{p+|l|}{p-k} \frac{t^k}{k!} = \binom{p+|l|}{p} + O(t), \tag{S2}$$

indicating that the beam profile remains nearly unchanged. Meanwhile, the derivative of the intensity is expressed as

$$\frac{\partial I}{\partial t} = \frac{I}{t} \left[|l| - t c_{lp}(t) \right], \tag{S3}$$

where

$$c_{lp}(t) = \begin{cases} 1, & p = 0, \\ 1 + 2L_{p-1}^{|l|+1}(t) \big/ L_p^{|l|}(t), & p > 0. \end{cases} \tag{S4}$$

Eqs. (S1)-(S3) show that within the ROI of MINFLUX, neither the beam intensity nor its derivative is significantly affected by the radial index. As a result, the CRB remains largely unchanged across different radial indices.

As an expansion of Eq. (4) in the main text, an analytical expression of the central CRB for MINFLUX with a nonzero radial index can be derived as

$$\sigma_0 = \frac{L}{2n\sqrt{2N}} \frac{s}{|l| - \frac{L^2}{2w^2} c_{lp}\left(\frac{L^2}{2w^2}\right)}. \tag{S5}$$

For a comprehensive demonstration, we calculate 2D CRB maps for MINFLUX with different p indices, as presented in Fig. S1.

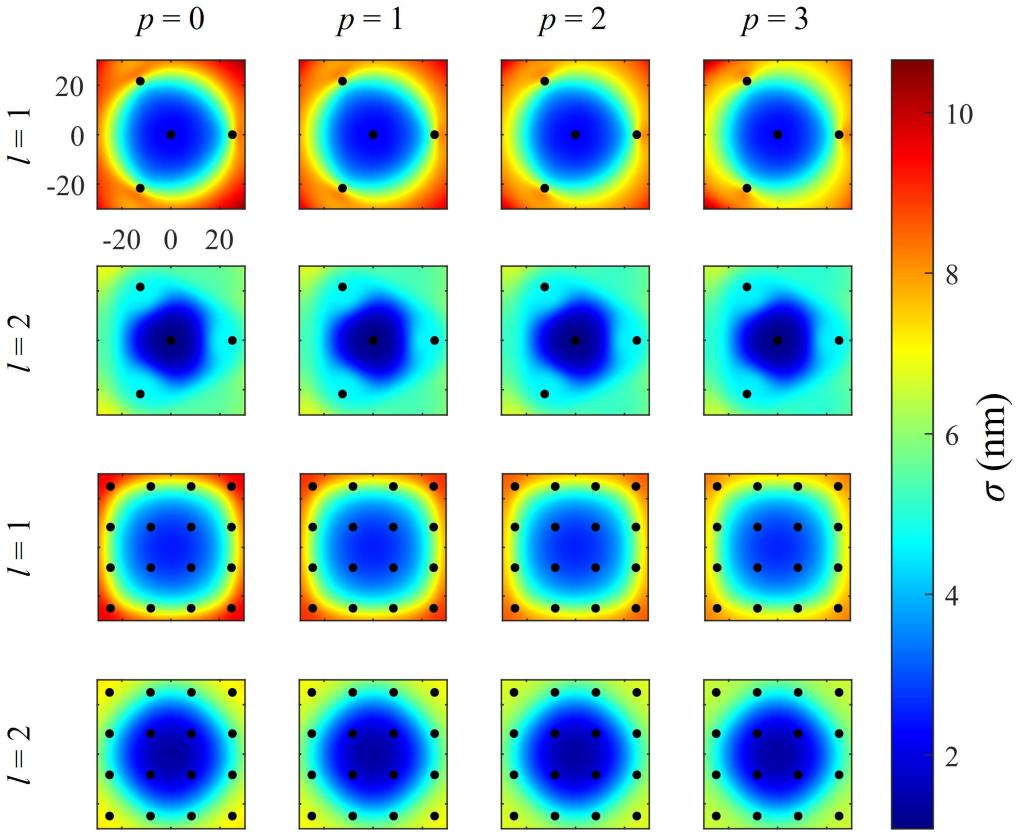

Fig. S1. The CRB of MINFLUX and RASTMIN with different radial indices. Other parameters are the same as in Fig. 3 of the main text.

As can be observed for both MINFLUX and RASTMIN, varying the radial index p from 0 to 3 results in minimal changes to the CRB, with only slight variations appearing at the edges of the FOV. In contrast, increasing the azimuthal index l significantly reduces the CRB, thereby leading to notable improvements in the localization precision. These results further support the inference that the radial index has a negligible impact on the localization precision of MINFLUX.

II. Anisotropy of CRB

The CRB of MINFLUX is generally not isotropic, and HO vortices further exacerbate this anisotropy. Following the definition in Ref.[1], the isotropy of the CRB is defined as

$$\mathbb{I} = \frac{\min(\sigma_1, \sigma_2)}{\max(\sigma_1, \sigma_2)}, \tag{S6}$$

where σ_1^2, σ_2^2 are the eigenvalues of the CRB matrix Σ_r.

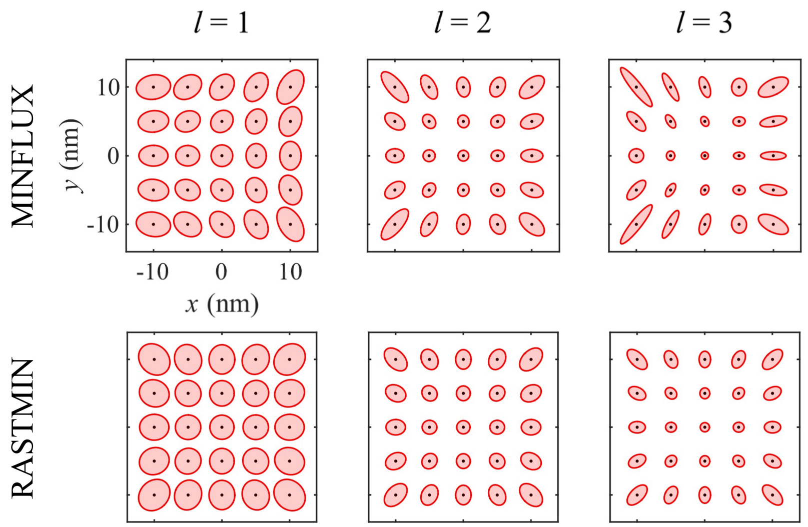

Fig. S2. Anisotropy of CRB in MINFLUX and RASTMIN. Parameters are the same as in Fig. 5 of the main text.

In Fig. S2, we present the anisotropy of CRB for MINFLUX and RASTMIN with different vortex orders. The CRB matrix is visualized as ellipses, with the lengths of their semi-axes representing the values of σ_1 and σ_2, and their orientations aligning with the corresponding eigenvectors. A circle at the center of the TCP indicates maximum isotropy $\mathbb{I} = 1$, indicating a uniform CRB in all directions. Our analysis reveals that the isotropy decreases as the vortex order l increases. For l = 1, 2 and 3, the average isotropy for MINFLUX is 0.72, 0.63 and 0.49, respectively. RASTMIN exhibits a similar trend, albeit with higher isotropy values given by 0.88, 0.75 and 0.67 for the same vortex orders. The decrease in isotropy primarily results from the CRB improving more significantly along certain directions as the vortex order increases, leading to a more anisotropic precision distribution.

III. Sensitivity to background noise

The primary analysis of MINFLUX in the main text focuses on different vortex beams under the same SBR conditions. To further elucidate the influence of background noise, we assess

the CRB for different vortex beams with an equal background intensity, and ensure the total beam intensities are also normalized accordingly.

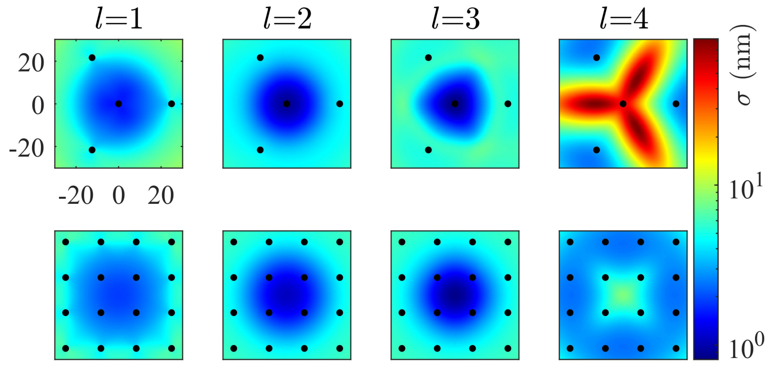

Fig. S3. The CRB for MINFLUX and RASTMIN under an equal background $\xi = 10^6$. The colormap is encoded in logarithmic scale to highlight variations. Other parameters are the same as in Fig. 3 of the main text.

We set an identical background intensity $\xi = 10^6$ for all vortex orders and calculate their corresponding CRBs, as presented in Fig. S3. Notably, while the CRB improves with increasing vortex order from $l = 1$ to $l = 3$, it deteriorates significantly when l increases to 4. This degradation arises because higher-order vortices exhibit lower SBR given the same background. For example, the central SBR for $l = 4$ drops below 0.1 for both MINFLUX and RASTMIN, making it impossible for any optical imaging. In comparison, vortex orders $l = 1$, 2, and 3 maintain SBR values exceeding 2, yielding improved precision with increasing vortex order.

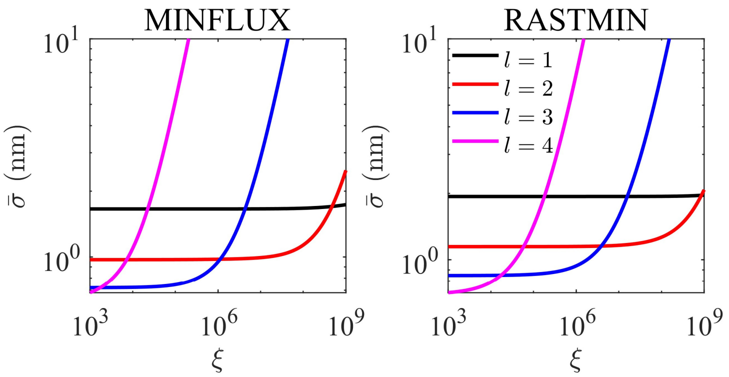

Fig. S4. Sensitivity of the CRB to background intensity for vortex beams with different orders.

Fig. S4 illustrates the mean CRB near the center versus background intensity for different vortex beams. Obviously, when the background is weak enough, higher-order vortices achieve superior localization precision. However, these beams are also more sensitive to background noise. As the background intensity increases, the CRB for higher-order vortices rises more rapidly compared to lower-order vortices. Once the background intensity surpasses critical levels where a high SBR no longer remains, the resulting CRB increases dramatically. For instance, in MINFLUX, higher-order vortex beams with orders $l = 2$, 3, and 4 become inferior to the first

order vortex beam when the background reaches 2.3×10^4, 4.4×10^6, and 4.7×10^8, respectively, corresponding to SBR values of 0.53, 0.67, and 1.18. All these turning points occur as the SBR drops below 2, suggesting that only at sufficiently high SBR levels do higher-order vortices outperform lower-order ones in MINFLUX.

Therefore, the superiority of HO vortices in MINFLUX strongly depends on maintaining a relatively high SBR. Properly increasing the intensity of vortices or suppressing the background noise can significantly enhance the applicability of HO vortex beams in MINFLUX.

IV. HO vortex generation with VPP

While HO vortex beams have been effectively generated using multiple techniques, here we show that they can be efficiently produced with HO VPPs in high NA imaging systems, which is fully compatible with conventional MINFLUX setups.

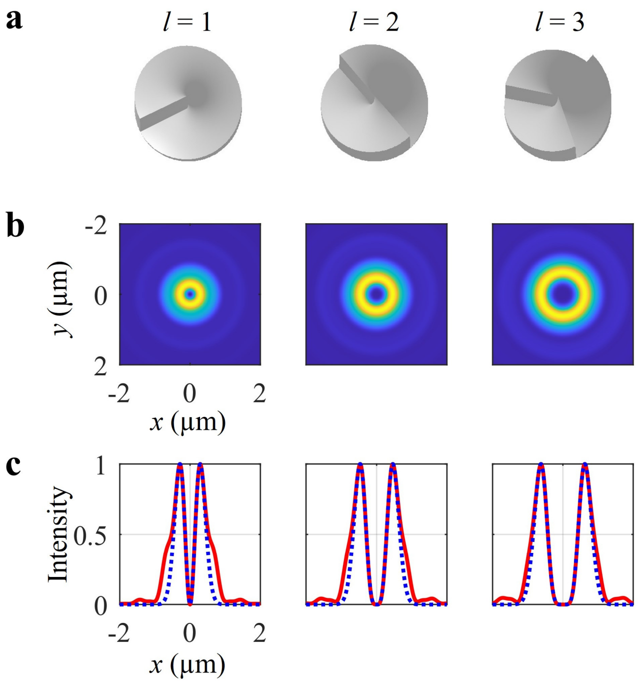

Fig. S5. Calculation and fitting of a circularly polarized light beam passing through a VPP and focused by a high NA objective lens. (a) VPPs with azimuthal indices $l = 1$, 2 and 3. (b) Corresponding beam profiles at the focal plane. (c) Radial intensity distribution of the focused beams (red, solid lines) compared to the fitted vortex beams (blue, dashed lines).

Consider a left-handed circularly polarized plane wave passing through a VPP and then focused by an objective lens, as is done in most MINFLUX configurations. Under high NA conditions (set as 0.9), the paraxial approximation does not hold. To accurately evaluate the focused beam

profile, we employ Richards–Wolf vectorial diffraction theory[2] to calculate the field distribution at the focal plane, as presented in Fig. S5.

As evident from our calculations, the focused beam closely approximates a vortex beam of corresponding order. Given the definition of mode fidelity as

$$\eta = \left\langle \sqrt{I_{foc}} \middle| \sqrt{I_{fit}} \right\rangle^2, \tag{S7}$$

where I_{foc} and I_{fit} are the normalized intensity profiles of the focused beam and fitted vortex beam. The fidelities obtained for l =1, 2, and 3 are 73.5%, 82.6% and 87.5%, respectively. Specifically, as MINFLUX primarily utilizes the central area of the beam, the fidelity exceeds 99% for all examined orders within the central 500 nm region, indicating a high-efficiency generation of HO vortex beams through VPPs.

It is worth noting that polarization plays a crucial role in shaping the focus of high NA systems. For instance, reversing the handedness of circularly polarized light in MINFLUX can dramatically alter the focal field distribution, transforming the dark-centered doughnut into a bright focal spot. Moreover, the focusing properties can be effectively manipulated using vector beams with spatially varying polarization[3]. These polarization-dependent effects are powerful for tailoring the PSF of imaging systems, offering an additional degree of freedom to optimize the precision of MINFLUX.

References

1 Balzarotti, F., Eilers, Y., Gwosch, K. C., Gynnå, A. H., Westphal, V., Stefani, F. D., Elf, J. and Hell, S. W. Nanometer resolution imaging and tracking of fluorescent molecules with minimal photon fluxes. *Science* **355**, 606-612 (2017).

2 Richards, B. and Wolf, E. Electromagnetic diffraction in optical systems, II. Structure of the image field in an aplanatic system. *Proceedings of the Royal Society of London. Series A. Mathematical and Physical Sciences* **253**, 358-379 (1959).

3 Zhan, Q. Cylindrical vector beams: from mathematical concepts to applications. *Advances in Optics and Photonics* **1**, 1-57 (2009).
